# Benefits of preoperative embolization before resection of a giant convexity meningioma: a case report

**DOI:** 10.11604/pamj.2024.48.108.44206

**Published:** 2024-07-17

**Authors:** Putri Arifin, Yudhi Adrianto, Djohan Ardiansyah

**Affiliations:** 1 Department of Neurology, Soetomo General Academic Hospital, Surabaya, Indonesia,; 2 Department of Neurology, Airlangga University Hospital, Surabaya, Indonesia,; 3 Department of Neurology, Faculty of Medicine, Airlangga University, Surabaya, Indonesia

**Keywords:** Embolization, meningioma, resection, case report

## Abstract

Meningiomas are typically benign and asymptomatic, but complicated or symptomatic cases may necessitate immediate intervention. Specifically, a giant meningioma with high vascularization poses a surgical challenge due to increased risk. In this report, a case of a giant meningioma managed with preoperative embolization (POE) before surgery was presented. A 24-year-old woman presented with severe bilateral visual disturbances and chronic headaches. Upon examination, magnetic resonance imaging (MRI) showed a giant meningioma measuring 97 mm in maximum diameter. Subsequent angiography indicated a significant tumor blush, primarily supplied by the right anterior cerebral artery (RACA) and the right middle cerebral artery (RMCA), with additional dural supply from the distal branches of the right and left middle meningeal artery (R-L MMA). Following this, endovascular embolization was performed, achieving 75% occlusion of the MMA using polyvinyl alcohol (PVA). The tumor was subsequently resected, attaining Simpson grade 1 on the fifth day post-embolization. Theoretically, preoperative embolization offered several advantages, including tumor devascularization, reduced operative blood loss, improved tumor visualization, and potentially higher rates of achieving Simpson grades I or II resection. The MMA was frequently targeted for embolization in meningioma cases, with no additional neurological deficit observed afterward. The optimal timing for resection typically fell within 1-7 days after embolization. During surgery, the patient experienced 1000 ml of bleeding over a 7-hour duration. The results showed that preoperative embolization provided significant benefits in reducing bleeding, shortening operating time, and facilitating mass resection, thereby affecting long-term recurrence rates, reported at 9% over 10 years.

## Introduction

In a 1922 publication, Harvey Cushing coined the term “meningioma” to describe a tumor that is originated in the meningeal (dural) region of the brain and spinal cord [1]. Intracranial meningioma is more prevalent in women, with a female-to-male ratio of 2: 1 [2]. Among all central nervous system tumors, meningioma and other mesenchymal tumor account for 27%, with an incidence rate of 6/100,000 (most commonly affecting individuals over 50 years of age) [3]. Although most are benign lesions, there is significant heterogeneity in histology, recurrence rate, aggressiveness, symptoms, and survival outcomes [4]. The exact cause of meningioma remains unknown, but deletion and inactivation of the neurofibromatosis 2 (NF2) gene locus, a tumor suppressor gene, are considered the predominant factors in sporadic meningioma [3]. A giant intracranial meningioma is defined as a meningioma within the cranium with a diameter exceeding 6cm [5]. The tumor differs from others due to its large size, which often leads to increased intracranial pressure and proximity to critical anatomical structures. The most common locations for a giant intracranial meningioma include the convexity, parasagittal/falcine region, sphenoid wing, tuberculum sellae, and posterior fossa. Clinical symptoms typically relate to the tumor's location within the skull or the presence of increased intracranial pressure.

Radiological diagnosis is usually delayed until the tumor has reached a considerable volume due to its slow growth. Additionally, a giant intracranial meningioma often has calcification on plain skull X-rays or appears to have bone changes [6]. This type of tumor is vascular-rich and prone to significant bleeding during mass resection. The main treatment method covers mass resection, supplemented by therapeutic modalities including chemotherapy and radiotherapy [4]. Mass resection is the primary objective of surgical management for a giant intracranial meningioma [6]. Surgery is customized to each tumor based on size and location to maximize effectiveness and safety during tumor resection. However, surgery for a giant intracranial meningioma poses challenges due to its large volume, high vascularization, and numerous neurovascular structures that may obscure tumor visualization and cause severe brain edema [5]. Despite surgical efforts, achieving a total mass resection is not always feasible in a giant intracranial meningioma, primarily due to its attachment to important neurovascular structures, such as the optic nerve, carotid artery, or superior sagittal sinus [6].

Several reviews have shown that preoperative embolization (POE) can improve the chance of achieving mass resection in a giant supratentorial meningioma. Conventional angiography is typically unnecessary unless embolization is planned, which is shown when the tumor is mainly supplied by the external carotid artery (ECA). The concept of preoperative embolization was first described by Manelfe at 1986, proving to be beneficial in reducing bleeding and shortening the operation time. Complications associated with preoperative embolization have been reported at rates ranging from 1.6% to 4.6%, with a mortality rate of less than 0.5%. In clinical practice, polyvinyl alcohol (PVA) particles are commonly used as embolization agents, with small particles (45-150 µm) carrying a higher risk of complications [7] The grade of resection, categorized by Simpson's grading criteria, has been identified as an important prognostic factor [1]. Most meningiomas are usually vascularized by branches of ECA, facilitating access for embolization [8]. Controversies persist regarding the indications for preoperative embolization, the interval between embolization and surgery, and the benefits of embolization before surgery. In this instance, a case report is presented detailing the treatment of a giant convexity meningioma treated with preoperative endovascular embolization before mass resection, resulting in clinical neurological improvement in the patient.

## Patient and observation

**Patient information:** a 24-year-old female presented with a gradual onset of blurred vision over the past four months. Initially, she experienced blurred vision in the left eye, which progressed to seeing only shadows or waving hands after one month. The patient further noticed a decrease in the vision in the right eye, which made her visit the nearest hospital where she was diagnosed with optic nerve swelling and referred to the neurology clinic. One month later, the patient reported complete loss of vision in both eyes but could still see people's shadows when they were in close proximity. Upon assessment in the clinic, she reported an absence of light perception in both eyes and also complained of chronic headaches persisting for the past year. The patient experienced frontal headaches, characterized by a feeling of tightness, without throbbing or radiation. The headaches occurred almost daily, lasting for 1-2 hours, with a numeric rating scale (NRS) score of 1-2. Furthermore, the headaches improved with paracetamol use and were not accompanied by weakness, tingling, or thickness on one side of the body. There were no reports of vomiting, seizures, or changes in behavior such as irritability or slurred speech. The patient denied any prior medical history, family history of similar complaints, and use of hormonal contraceptives.

**Clinical findings:** based on general examination, vital signs and general physical condition were found to be normal. Neurologically, the patient had isochoric and round pupils measuring 4mm/4mm, with negative light reflexes in both eyes. There was no light perception in either eye, and no other focal neurological deficits were identified. Funduscopic examination found atrophic optic discs in the left eye and edematous optic discs in the right eye.

**Figure 1:**
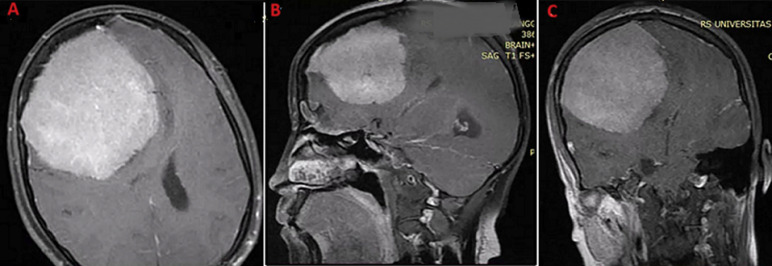
A) axial section on T1-contrast head magnetic resonance imaging showing a hyperintense lesion of giant convexity meningioma; B) sagittal; C) coronal

**Diagnostic assessment:** the magnetic resonance imaging magnetic resonance imaging (MRI) of the head with contrast showed a hyperintense lesion in the right frontoparietal region measuring approximately 9.7 x 7.5 x 7.6cm, accompanied by perifocal edema and frontal hyperostosis. The right anterior cerebral artery (RACA) was identified as the primary blood supply for the mass, with bilateral optic nerve atrophy observed (Figure 1).

**Therapeutic intervention:** embolization started with an anterograde puncture of the right femoral artery, followed by the insertion of a 5F femoral sheath. A total of 100 cc of contrast was administered, and using 4-dimensional digital subtraction angiography (DSA) and bilateral ECA catheterization, the dural feeder, pial supply of the tumor, and venous drainage system were identified. After assessing harmful anastomoses and confirming the dominant supply from ECA, it was determined that the tumor had pial supply from the distal branches of the RACA and right middle cerebral artery (RMCA), as well as dural supply from distal branches of the right and left middle meningeal artery (R-L MMA). Subsequently, the tumor feeder was catheterized using Vasco 10 and Hybrid 008 microcatheter-microwire, followed by embolization with PVA 150 and 300 in series, achieving 75% devascularization. The patient remained stable throughout and after the cerebral angiography procedure, with no new neurological deficits observed. Following embolization, the patient experienced improved vision in the left eye to 1/300. Resection was performed on the fifth day after embolization, achieving mass resection with minimal bleeding of approximately 1000ml over a 7-hour operation, graded as Simpson grade I. Postoperative management was successful, resulting in a remarkable improvement in visual acuity, and the patient was discharged nine days after surgery. Anatomical pathology analysis showed the tumor tissue as transitional and metaplastic meningioma, classified to be World Health Organization (WHO) grade I.

**Follow-up outcomes:** at an 11-month follow-up post-resection, the patient had improved vision in the right eye to 1/60.

**Patient perspective:** the patient declined to give his perspective on her illness.

**Informed consent:** written informed consent was obtained from the patient for publication and any accompanying images.

## Discussion

Preoperative embolization continued to play an important role in the management algorithms of meningioma. Neurointervention in our country constituted a therapeutic method necessitating the presence of DSA facilities, specialized hardware, trained laboratory technicians, and skilled nursing personnel, which were still limited to select centers across the country, including major cities. In certain cases with a successful outcome, embolization could serve as the primary therapeutic modality, obviating the need for additional surgery [8]. Preoperative tumor embolization along with comprehensive vascular devascularization was performed to minimize blood flow to the tumor. Tumor location played an important role in determining prognosis and treatment options, particularly regarding surgical resectability. However, the true biological and radiological profile of meningioma, as well as the diverse outcomes experienced by a patient receiving surgical treatment for this rare condition, remained unclear. The majority of meningiomas were found in the supratentorial compartment, commonly along the dural venous sinuses in the cerebral convexity, parasagittally, and in sphenoid wing regions [4]. Hypervascular meningioma frequently had a characteristic “sunburst” appearance on neuroimaging, signifying a large arterial pedicle and the vascular branches radiating out from the feeding artery [9]. In the short term, tumor embolization might have direct effects such as apoptosis and reduced tumor growth. Meanwhile, in the long term, it could lead to paradoxical effects, particularly malignant transformation due to tumor hypoxia.

**Figure 2:**
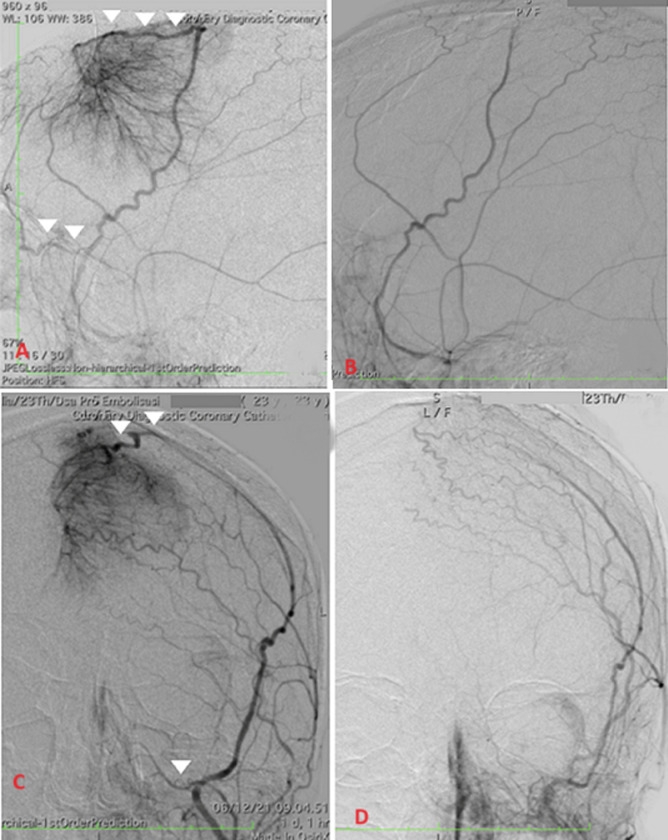
angiogram of the right middle meningeal artery, lateral view; A) before and B) after embolization; angiogram of the left middle meningeal artery, anterior view, C) before and D) after embolization

This showed the timing following tumor embolization might influence the molecular biological impacts, necessitating further reviews to fully understand the time-dependent phenomena [2]. Based on previous study, preoperative angiography and embolization were recommended under the following conditions: i) tumor >3-4 cm in diameter, with at least 50% of the supply originating from accessible branches of the ECA; ii) tumor that appeared hypervascular or had a deep-seated vascular supply difficult to access surgically based on noninvasive neuroimaging; iii) tumor located in eloquent areas iv) tumor without extensive calcification, except in certain circumstances [2]. Convexity meningioma having multidirectional blood flow and an outward appearance of being hypervascular were considered the best candidates for embolization, although there were no set anatomic location criteria for selecting candidates [2]. In this case, a successful closure of three out of four right-left MMA branches was achieved. (Figure 2). The primary benefits of embolization therapy arose from effectively blocking the vascularization of large tumors, with a significant success rate observed when targeting the intracranial tumor supply. PVA particles have been the preferred embolic agent in most reviews on meningioma embolization. These particles tended to aggregate and could occlude blood vessels larger than their diameter.

To ensure safety during the embolization process, PVA particles larger than 100 µm were used [7]. Despite its benefits, embolization carried certain risks, such as large vessel dissection, microcatheter fracture, and unintended occlusion of arteries or veins, which resulted in infarction or bleeding. In a study engaging 185 cases of preoperative embolization, 12 patients (7%) experienced procedure-related complications, including ischemia and bleeding. They concluded that the individual risk-to-benefit ratio of preoperative embolization needed to be considered in clinical decision-making. Reported complications from preoperative embolization included cranial nerve deficits in 2 cases out of 80 cases, with no permanent neurological complications observed in the remaining patients. Minor post-procedure complications were reported in 10% of cases, including transient localized femoral hematoma. Moreover, only one case of post-operative skin necrosis was reported, which resolved within 3 weeks with routine bandaging [2]. The three surgical resection steps for meningioma, including devascularization, *debulking*, and dissection, could be facilitated more effectively when tumor blush was totally or subtotal reduced [2]. Bleeding during meningioma resection posed a significant risk, which was crucial to consider when evaluating treatment options. Preoperative embolization might be considered as an additional therapy to make meningioma resection easier with fewer complications [2]. Intraoperative bleeding represented a major complication in brain tumor surgery and was directly related to patient morbidity and mortality. The average intraoperative bleeding ranged from 200ml to 2.2L, often requiring blood transfusion for a patient [2].

**Figure 3:**
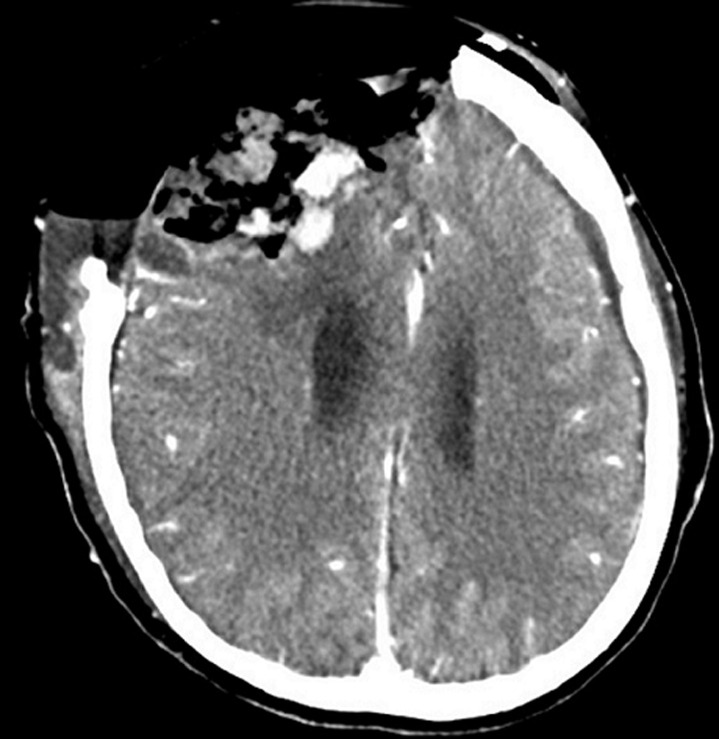
contrast-enhanced computer tomography image on axial section post-resection: no residual mass is visible

The extent of resection was based on various factors, including the lesion's location, size, and proximity to vital structures [10]. Some analysts suggested that resection needed to be performed within 1-7 days after embolization to avoid revascularization [7] which might render the tumor softer, thereby facilitating *debulking* and easing tumor capsule dissection [2]. Patients with WHO grade I meningioma had a better survival prognosis with a recurrence rate of 9% within 10 years [1]. In this case, mass resection according to Simpson grade 1 was successfully achieved and periosteal dura grafting was performed (Figure 3). Devascularization through preoperative embolization was considered to enhance tumor control by reducing intraoperative bleeding and operation time, and potentially extending the time to recurrence. Additionally, the average treatment cost was lower in the embolized group, showing potential cost-effectiveness. Further reviews were required to investigate the tumor control effect of preoperative embolization in challenging resection cases. The benefits of embolization were influenced by various factors, including tumor characteristics, radiological features, and surgical aggressiveness. As clinicians, it was important to carefully weigh the benefits of embolization against the procedural risks.

## Conclusion

The case report details the successful treatment of a 24-year-old woman presenting with severe visual disturbances and chronic headaches caused by a giant meningioma. The tumor, located in the right frontoparietal region, posed a surgical challenge due to its large size and high vascularization. The authors opted for preoperative embolization to revascularize the tumor before resection surgery. The embolization procedure achieved significant devascularization, leading to reduced intraoperative bleeding and successful mass resection with minimal complications. The patient experienced improved vision post-embolization and continued to show improvement at the 11-month follow-up. The manuscript underscores the importance of preoperative embolization in reducing intraoperative bleeding, shortening operation time, and facilitating successful tumor resection, especially in cases of giant, highly vascularized meningiomas. The authors discuss the theoretical advantages of POE, including tumor devascularization, improved visualization during surgery, and potentially higher rates of achieving Simpson grades I or II resection. They also highlight the need for careful patient selection and consideration of the risks associated with embolization. Overall, the manuscript supports the efficacy of preoperative embolization as adjunctive therapy in the management of giant intracranial meningiomas, offering potential benefits in terms of surgical outcomes and long-term recurrence rates.
